# CXCL13 expression in mouse 4T1 breast cancer microenvironment elicits antitumor immune response by regulating immune cell infiltration

**DOI:** 10.1093/pcmedi/pbab020

**Published:** 2021-08-04

**Authors:** Qizhi Ma, Yue Chen, Qing Qin, Fuchun Guo, Yong-sheng Wang, Dan Li

**Affiliations:** Department of Thoracic Oncology, State Key Laboratory of Biotherapy and Cancer Center, West China Hospital, Sichuan University, Chengdu 610041, China; Department of Thoracic Oncology, State Key Laboratory of Biotherapy and Cancer Center, West China Hospital, Sichuan University, Chengdu 610041, China; Department of Thoracic Oncology, State Key Laboratory of Biotherapy and Cancer Center, West China Hospital, Sichuan University, Chengdu 610041, China; Institute of Drug Clinical Trial, State Key Laboratory of Biotherapy and Cancer Center, West China Hospital, Sichuan University, Chengdu 610041, China; Institute of Drug Clinical Trial, State Key Laboratory of Biotherapy and Cancer Center, West China Hospital, Sichuan University, Chengdu 610041, China; Institute of Respiratory Health, Frontiers Science Center for Disease-related Molecular Network, and Precision Medicine Center, Precision Medicine Key Laboratory of Sichuan Province, West China Hospital, Sichuan University, Chengdu 610041, China

**Keywords:** CXCL13, triple negative breast cancer, 4T1, tumor microenvironment, CXCR5

## Abstract

Breast cancer is the most commonly diagnosed cancer type and the leading cause of cancer-related deaths among women worldwide. Previous studies have reported contradictory performance of chemokine CXC motif ligand 13 (CXCL13) in breast cancer. In this study, The Cancer Genome Atlas database analysis revealed that CXCL13 was overexpressed in various human cancers including breast carcinoma, and associated with good clinical prognosis in breast cancer. Flow cytometry detection also found upregulated intracellular CXCL13 expression in human breast cancer cell lines. To explore the possible role of CXCL13 in the breast cancer microenvironment, mouse triple negative breast cancer (TNBC) was lentivirally transfected to stably overexpress mouse CXCL13 (4T1-CXCL13). Both parental 4T1 and 4T1-CXCL13 strains showed no *in vitro* or *in vivo* endogenous cell surface CXCR5 expression. In immune-competent BALB/c mice, the *in vivo* tumor growth of 4T1-CXCL13 was significantly inhibited and even completely eradicated, accompanied with increased infiltrations of CD4^+^, CD8^+^ T lymphocytes and CD11b^+^CD11c^+^ DCs. Further investigations showed that CXCL13 expression in the 4T1 tumor microenvironment elicited long-term antitumor immune memory, and rejection of distal parental tumor. The antitumor activity of CXCL13 was remarkedly impaired in BALB/cA-nu nude mice, or in BALB/c mice with CD8^+^ T lymphocyte or NK cell depletion. Our investigation indicated that CXCL13 expression in TNBC triggered effective antitumor immunity by chemoattracting immune cell infiltrations and could be considered as a novel prognostic marker for TNBC.

## Introduction

As the leading cause of cancer-related death in women, breast cancer has ranked the first in incidence rate of all female cancer patients worldwide,^1^ and triple-negative breast cancer (TNBC) is the most aggressive and metastatic pathological type. Lacking expression of estrogen receptor, progesterone receptor, and type II human epidermal growth factor receptor, TNBC accounts for ∼10%–20% of all pathological types of breast cancer.^2^ Compared with other breast cancer types, TNBC patients have significantly higher 5-year recurrence rate, are more prone to tumor metastasis, and have a shorter survival time after metastasis and higher mortality.^3^ Currently, there is no specific treatment for TNBC; chemotherapy is still the only choice for adjuvant therapy or tumor metastasis, and only 30% of patients respond to chemotherapy.^4^

Chemokine CXC motif ligand 13 (CXCL13), also called B lymphocyte chemokine or B cell-attracting chemokine 1, belongs to the CXC chemokine family. Under normal physiological conditions, by acting on its receptor CXCR5, CXCL13 plays a pivotal role in germinal center formation, B cell attraction, as well as chemotaxis and differentiation of plasma cells and memory B lymphocytes.^5^ In addition, CXCL13 has also been reported to exert important functions in the pathogenesis of autoimmune and lymphoproliferative diseases.^6–8^ For instance, CXCL13 has been reported to attract and maintain B and T cells in the inflamed central nervous system lesions in multiple sclerosis patients.^9^

In recent years, several studies have reported the involvement of CXCL13 in the progression, metastasis, and prognosis of solid tumors,^10^ including breast cancer,^11^ colon cancer,^12^ prostate cancer,^13^ renal cell carcinoma,^14^ penile cancer,^15^ gastric cancer,^16^ etc. Compared with breast cancer tissues, CXCL13 is expressed at a low level in normal breast or adjacent noncancerous tissues.^17,18^ Notably, conflicting opinions have been raised regarding the role of CXCL13 in breast cancer. On one hand, in a large cohort study (1010 cases) of early breast cancer patients conducted by Schmidt et al., total RNA was extracted from formalin-fixed paraffin-embedded (FFPE) tumor sections, and CXCL13 expression was evaluated using quantitative polymerase chain reaction (qRT-PCR). Breast cancer CXCL13 expression exhibited an independent influence on patients and was associated with favorable distant disease-free survival, especially in TNBC.^19^ Furthermore, Criscitiello et al. found that in TNBC, a four-gene signature including CXCL13 predicted high levels of tumor infiltrating lymphocytes (TILs) after neoadjuvant therapy and was associated with good clinical outcome.^20^ Likewise, Christina et al. reported that a novel 14-gene panel containing CXCL13 predicted longer metastasis-free survival in early stage hormone receptor-negative breast cancer and TNBC.^21^ On the other hand, CXCL13–CXCR5 coexpression on breast cancer has been found to be correlated with tumor lymph node metastasis (LNM). Biswas et al. found that in 98 breast cancer patients diagnosed with infiltrating duct carcinoma, coexpression of CXCL13 and CXCR5 was significantly associated with LNMs, and after CXCL13 stimulation, various mesenchymal markers as well as epithelial–mesenchymal transition (EMT) regulators were markedly increased in CXCR5-transfected human breast cancer cell lines including MDA-MB-231.^22^ Despite this, Pimenta et al. found that after transfection with interferon regulatory factor 5, the transcription factor of CXCL13, human breast cancer cell MDA-MB-231 showed enhanced chemoattraction of healthy donor CXCR5 + T and B cells.^23^

In the current study, to investigate the potential function of CXCL13 in the TNBC microenvironment, a CXCR5-negative mouse 4T1 cell was lentivirally transfected to overexpress CXCL13 (4T1-CXCL13). In immune-competent BALB/c mice, the *in vivo* tumor growth of 4T1-CXCL13 was significantly inhibited and even completely eradicated, accompanied with increased infiltrations of CD4^+^, CD8^+^ T lymphocytes, and CD11b^+^CD11c^+^ DCs. Further investigations showed that CXCL13 mediated long-term antitumor immune memory and rejection of distal parental tumor. Our results suggested that CXCL13 expression in the TNBC environment mediated antitumor immunity, and provided new insight into the role of CXCL13 in TNBC progression.

## Materials and methods

### Expression of CXCL13 in tumors from TCGA Database

The expression of CXCL13 in 9498 tumors (including 31 subtypes) and 5540 normal samples from the The Cancer Genome Atlast (TCGA) database and the Genotype-Tissue Expression (GTEx) project were analyzed using the online Gene Expression Profiling Interactive Analysis (GEPIA) web server (http://gepia.cancer-pku.cn). The correlation between CXCL13 expression and cancer prognosis was investigated. The disease-free survival (DFS) was analysis by GEPIA. Data were downloaded, filtered for primary tumors, log2-transformed, and analyzed using R.

### Cell culture

Human breast cancer cell lines (MDA-MB-231, MDA-MB-468, and BT474), mouse breast cancer cell 4T1, and human embryonic kidney (HEK) 293T cells were obtained from the American Type Culture Collection. MDA-MB-231, MDA-MB468, BT474, and HEK 293T were cultured in DMEM (Gibco), and 4T1 was cultured in RPMI-1640 (Gibco). All the above media were supplemented with 10% fetal bovine serum (Invitrogen Life Technologies), 100 U/ml penicillin, and 100 *μ*g/ml streptomycin (Sangong Biotech). The cells were maintained in a humidified chamber at 37°C in 5% CO_2_ atmosphere.

### Lentivirus production

The cDNA sequence of murine CXCL13 (pORF-CXCL13) was purchased from the InvivoGen Company. CXCL13 gene was amplified by PCR using primers (forward: 5′-TGCTCTAGAATGGAGACAGACACACTCCTG-3′, reverse: 5′-GAGTAAGAGAAGAGCTGCCTGAGAATTCCGG-3′), including 5′ Xbal I and 3′ EcoR I sites, and then cloned into a lentiviral vector pCDH-CMV-MCS-Puro (pCDH-CXCL13). HEK-293T cells were transfected with the lentivirus plasmid pCDH-CXCL13, and two packaging plasmids pMD2.G and psPAX2 (kindly provided by Dr. Yan Luo, Sichuan University) at a ratio of 2:1:2 by TransIT 2020 transfection reagent (Mirus). The empty vector (pCDH) was also packaged as a negative control. Culture supernatants were collected at 48 and 72 h after transfection and filtered by 0.45 *μ*m filters.

### Generation of CXCL13-expressing 4T1 cells

CXCL13-expressing 4T1 stable cell strains were generated via lentivirus transduction, followed by 2 weeks selection of 3 *μ*g/ml puromycin (Sigma-Aldrich). Selected cell clones were seeded onto six-well plates at a density of 2 × 10^5^ cells/well and in 2 ml of RPMI-1640 complete medium. Forty-eight hours later, cell culture supernatant was harvested, and CXCL13 secretion was detected by Quantikine Mouse CXCL13 Immunoassay (MCX130, R&D), following the manufacturer's recommendations.

### EdU assay

*In vitro* cell proliferation was measured using the EdU (5-ethynyl-2′-deoxyuridine) assay (RIBOBIO). EdU is incorporated as thymidine analog into DNA during cell proliferation, and can be detected by a copper-catalyzed reaction with Apollo 643 dye (red fluorescence). Briefly, cells (5 × 10^3^ per well) were seeded in 96-well plates in RPMI-1640 with 10% fetal bovine serum and allowed to adhere overnight. EdU labeling was done by incubating with 50 M EdU for 2 h and then fixing in 4% paraformaldehyde. After washing with 2% glycine solution, cells were incubated with Apollo for 30 min, washed by 0.5% Triton X-100, and counterstained with Hoechst 33342. The percentage of EdU-positive cells was calculated at 100× magnification with nine randomly chosen fields, and graphed by a Thermo Cellomics Array scan VTI HCS Reader.

### *In vivo* tumor growth

BALB/c and BALB/cA-nu mice (female, 6–8 weeks old) were purchased from the Beijing HFK Bioscience Co. Ltd. All procedures were approved by the Institutional Animal Care and Use Committee of Sichuan University. Tumor cells were harvested at the logarithmic growth phase, and mice were subcutaneous (s.c.) inoculated in the right dorsal flank with 2 × 10^5^ parental 4T1, 4T1-pCDH, 4T1-CXCL13-1, or 4T1-CXCL13-2 cells in 0.1 ml phosphate-buffered saline (PBS). Each group contained 10 mice. Tumor volume was measured every 3 days, and calculated as length × width^2^ × 0.5. For ethical reasons, experiments were terminated when tumor volume reached 2000 mm^3^.

### Tumor rechallenge and bilateral inoculation tumor model

Six months after complete tumor regression, mice from 4T1-CXCL13-1 (*n* = 3) and 4T1-CXCL13-2 (*n* = 9) groups were rechallenged with s.c. injection of 2 × 10^5^ parental 4T1 cells. For the control group, naïve BALB/c mice (*n* = 6) were inoculated s.c. with 2 × 10^5^ parental 4T1 cells. Tumor volume was monitored and calculated every 3 days. For bilateral inoculation experiment, the first group of mice (*n* = 8) were inoculated s.c. in the left dorsal flank with 2 × 10^5^ 4T1-CXCL13-2 cells, and in the right dorsal flank with 2 × 10^5^ parental 4T1 cell. The second group of mice (*n* = 8) were inoculated s.c. in the left dorsal flank with 2 × 10^5^ 4T1-pCDH cells, and in the right dorsal flank with 2 × 10^5^ parental 4T1 cell. Parental 4T1 tumor volume was measured and recorded every 3 days, and calculated as length × width^2^ × 0.5. For ethical reasons, experiments were terminated when tumor volume reached 2000 mm^3^. Parental 4T1 tumor volume was measured and recorded every 3 days.

### Depletion of immune cell subsets *in vivo*

Immune cell depletions were performed as previously reported.^24^ Briefly, BALB/c mice were injected intraperitoneally (i.p.) at 100 *μ*g per dose with one of the following antibodies: anti-mouse CD4 ( 100442, BioLegend), anti-mouse CD8a ( 100746, BioLegend), or anti-mouse NK ( 146002, BioLegend). Mice were s.c. inoculated with 2 × 10^5^ 4T1, 4T1-pCDH, or 4T1-CXCL13-2 cells at day 0. Depleting antibodies were given on days –1, 1, 7, and 14. Tumor volume was measured and recorded every 3 days.

### Flow cytometry

For *in vitro* cell surface CXCR5 staining, 4T1 cells were harvested and incubated with PerCP-conjugated antimouse CXCR5 antibody (FAB6198C, R&D systems) or isotype PerCP Rat IgG2a (400 529, BioLegend). For *in vivo* 4T1 cell surface CXCR5 staining, samples were prepared as previously reported.^25^ Briefly, 5 × 10^5^ 4T1 cells were inoculated in the right dorsal flank of BALB/c mice, and tumors were obtained at 1 week after injection. Tissues were cut into small pieces, and digested into single-cell suspension by incubating in RPMI-1640 containing 1 mg/ml collagenase (Gibco). Then cell surface CXCR5 staining was performed as mentioned above.

For intracellular human CXCL13 staining, 4 *μ*l GolgiStop (containing monesin) was added in every 6-ml cell culture, and incubated at 37°C for 4 h. Then cells were harvested, intracellular staining was performed using fixation/permeabilization buffer (eBioscience), and incubated with goat antihuman CXCL13 antibody (AF801, R&D systems) or normal goat IgG control (AB-108-C, R&D systems), followed by Alexa Fluor 488-labled rabbit antigoat IgG secondary antibody (A11078, invitrogen) according to the manufacturer's instructions.

For 4T1 tumor-specific antibody detection, the blood of each group of BALB/c mice was collected from the orbital venous plexus at 4 weeks after tumor cell inoculation. Parental 4T1 cells were incubated with mouse serum (1:100 dilution) or PBS at 4°C for 30 min, and then stained with FITC-labeled goat antimouse antibody IgG.

For detection of intratumoral immune cell infiltration, tumor tissues were harvested at 14 and 21 days after inoculation, cut into small pieces, and then digested into single-cell suspension by incubating in RPMI-1640 containing 1 mg/ml collagenase (Gibco) at 37°C for 1–2 h. The following BD Pharmingen antibodies were used: CD4-PE, CD8a-FITC, CD11b-FITC, and CD11c-PE. Flow cytometry was performed on FACScan flow cytometer (BD FACSCalibur), and data were analyzed using Flowjo7.6.1.

### Immunohistochemistry and immunofluorescence staining

Tumor tissues were harvested at necropsy. For Ki-67 staining, FFPE tumor sections were dewaxed, and incubated overnight at 4°C with anti-Ki67 (ab16667, Millipore) primary antibodies after heat-induced epitope retrieval. For CXCL13, CD31, and IgG staining, tumor frozen sections were incubated overnight at 4°C with goat antimouse CXCL13 (AF470, R&D systems), rabbit antimouse CD31 (ab182981, abcam) primary antibodies or HRP-labeled goat antimouse IgG antibody (ab6728, abcam). Rabbit antigoat and goat antirabbit SABC-HRP and DAB reagent kits were purchased from the Beijing Zhongshan Jinqiao Biotechnology Co., Ltd. For immunofluorescence staining, tumor tissues were snap-frozen and cut into 8-*μ*m sections, and stained with antimouse CD4-FITC antibody (GTX44531, GeneTex) or antimouse CD8-FITC antibody (GTX74773, GeneTex).

### Hematoxylin and eosin staining

To evaluate pathological changes, mouse major organs were obtained at necropsy and fixed in 4% paraformaldehyde. Hematoxylin and eosin (H&E) staining was performed in FFPE tissue sections.

### Statistical analysis

All data were expressed as mean ± SD or mean ± SEM, and statistically analyzed by GraphPad Prism 8.0. Student's *t* test and analysis of variance were conducted to determine the statistical significance of experimental results. The findings were considered as significant if *P* < 0.05.

## Results

### CXCL13 expression is upregulated in various human cancers including breast carcinoma

**Figure 1.**
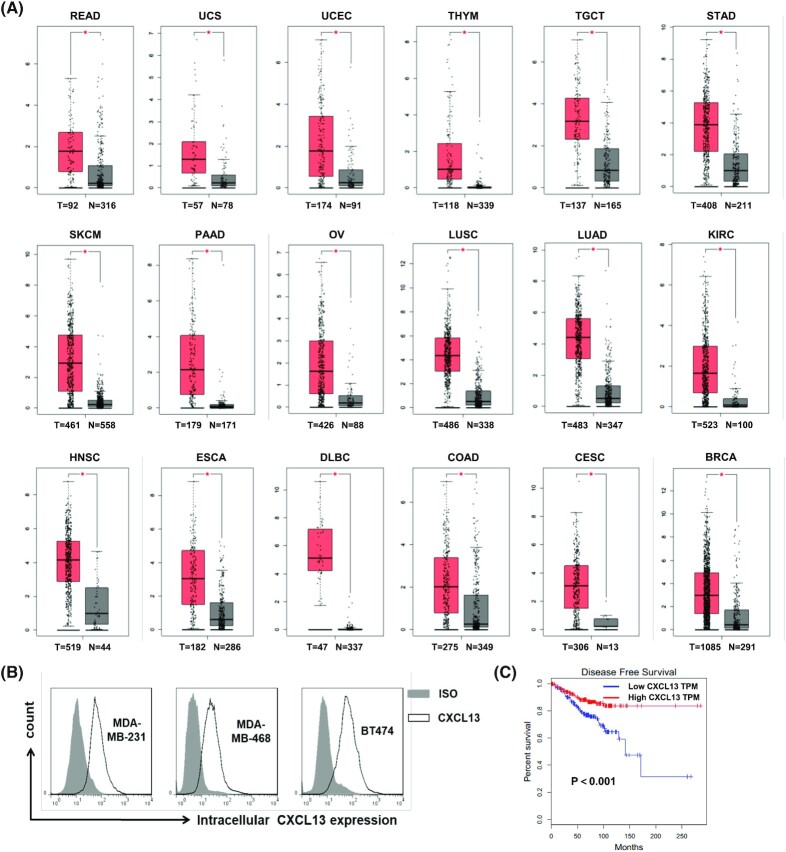
The expression of CXCL13 was upregulated in various human tumors including breast cancer. (A) The mRNA expression level of CXCL13 in the human cancers and normal tissues was analyzed using the online web server GEPIA. CXCL13 was significantly upregulated in rectum adenocarcinoma (READ), uterine carcinosarcoma (UCS), uterine corpus endometrial carcinoma (UCEC), thymoma (THYM), testicular germ cell tumors (TGCTs), stomach adenocarcinoma (STAD), skin cutaneous melanoma (SKCM), pancreatic adenocarcinoma (PAAD), ovarian serous cystadenocarcinoma (OV), lung squamous cell carcinoma (LUSC), lung adenocarcinoma (LUAD), kidney renal clear cell carcinoma (KIRC), head and neck squamous cell carcinoma (HNSC), esophageal carcinoma (ESCA), lymphoid neoplasm diffuse large B-cell lymphoma (DLBC), colon adenocarcinoma (COAD), cervical squamous cell carcinoma and endocervical adenocarcinoma (CESC), breast invasive carcinoma (BRCA); (T = tumor number, N = normal number, *P < 0.05). (B) The expression of CXCL13 in human breast cancer cell lines was detected by flow cytometry. (C) Correlational analysis between disease-free survival and CXCL13 expression level in BRCA through Kaplan–Meier analysis by GEPIA. Each point represents a different TCGA sample.

To confirm the expression of CXCL13 in human tumors, normalized mRNA levels of CXCL13 in human tumors and normal tissues were analyzed using the GEPIA web server. The RNA sequencing (RNA-Seq) expression data for 9498 tumors and 5540 normal samples from the TCGA and GTEx projects were included. As shown in Fig. 1A, compared with the normal tissues, CXCL13 expression was significantly upregulated in 18 of 31 tumor types, including breast invasive carcinoma. Furthermore, the intracellular CXCL13 were detected in human breast cancer cell lines MDA-MB-231, MDA-MB-468, and BT474 by flow cytometry, and all tumor cells had high expression levels of CXCL13 (Fig. 1B). In addition, the correlation between tumor tissue expressed CXCL13 and clinical survival was analyzed using the TCGA database. As a result, breast cancer patients with higher CXCL13 expression showed a significantly longer DFS (Fig. 1C).

### Generation of mouse TNBC 4T1 cells that stably express CXCL13

**Figure 2.**
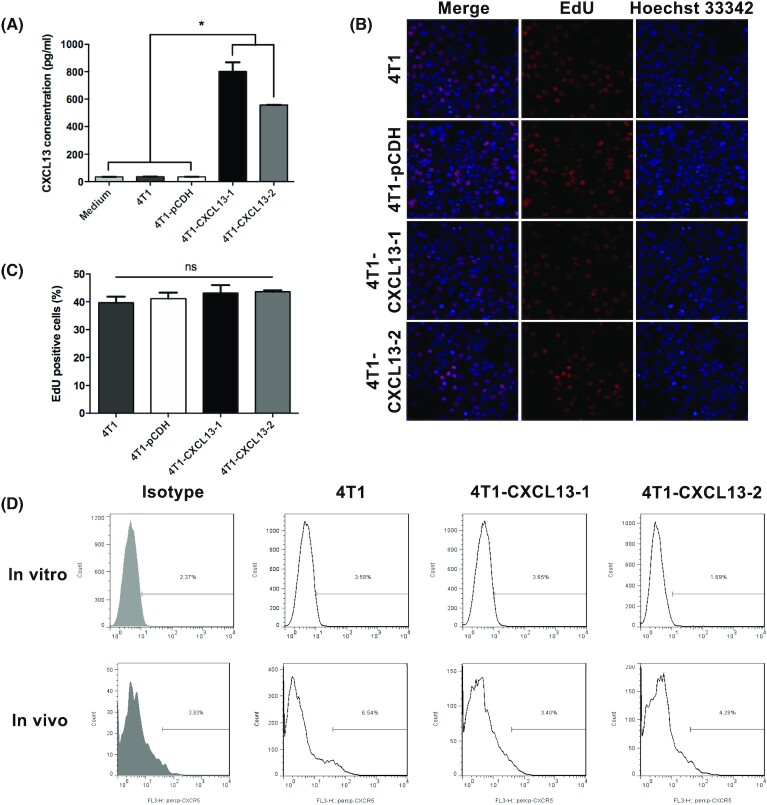
*In vitro* and *in vivo* proliferations and CXCR5 expression of mouse TNBC 4T1 that was transfected to stably overexpress CXCL13. (A) Mouse 4T1 cell was transfected with mouse CXCL13-expressing lentivirus, and cell clones 4T1-CXCL13-1 and 4T1-CXCL13-2 were screened for the following experiments. Cell culture supernatant detection showed that CXCL13 secretion was significantly elevated in 4T1-CXCL13-1 and 4T1-CXCL13-2 groups, compared to culture medium (CM), parental 4T1 and empty virus-transfected 4T1 (4T1-pCDH) groups. Bars, means ± SD (*n* = 3). (*P < 0.05). (B) Representative pictures of *in vitro* cell proliferation detected by EdU incorporation and Hoechest 33342 staining (magnification, ×200). (C) Statistical analysis of EdU positive cells in each group. Bars, means ± SD (*n* = 3). (D) Cell surface CXCR5 expression of 4T1 was detected both *in vitro* and *in vivo* by flow cytometry.

Mouse TNBC 4T1 that expressed mouse chemokine CXCL13 was constructed based on a lentiviral vector pCDH-CXCL13, and the stably expressed cell stains were screened by 3 *μ*g/ml puromycin. To confirm the successful construction, CXCL13 secretion in cell culture supernatant was detected by enzyme-linked immunosorbent assay. As a result, parental 4T1 showed no significant CXCL13 secretion, compared with the culture medium and empty vector-transfected 4T1 (4T1-pCDH). Two cell strains, 4T1-CXCL13-1 and 4T1-CXCL13-2, which had high expression levels of CXCL13, were selected for subsequent experiments (Fig. 2A).

### *In vitro* proliferations of 4T1-CXCL13 stably cell strains

To assess the impact of CXCL13 expression on 4T1 cell proliferation *in vitro*, EdU assay was performed. As shown in Fig. 2B, EdU was incorporated into newly synthesized cell DNA and stained with Apollo 643 dye (red). DNA was staining with Hoechest 33342 (blue), and purple cells showed EdU/Hoechest double-positive cells. The quantification of cell proliferation was conducted by statistical analysis of EdU-positive cells, and no significant difference was observed between each group (Fig. 2C). The result indicated that CXCL13 had no direct influence on 4T1 cell proliferation *in vitro*.

Next, the cell surface expression of CXCL13 receptor CXCR5 was assessed. Compared with the isotype control, parental 4T1, 4T1-CXCL13-1, and 4T1-CXCL13-2 cells showed no obvious CXCR5 expression *in vitro*. Previous study reported that the cell surface CXCR5 could be induced to upregulate on cancer cells after the *in vivo* engraftment, and quickly dropped after *ex vivo* culture.^25^ Based on this consideration, tumor tissues were obtained at 1 week after s.c. inoculation in BALB/c mice, and tumor cells were isolated by collagenase digestion for CXCR5 surface staining. Flow cytometry showed that there was no obvious CXCR5 expression *in vivo* implanting parental 4T1, 4T1-CXCL13-1, or 4T1-CXCL13-2 cells, compared with that of the isotype control (Fig. 2D). Together, these results indicated mouse TNBC 4T1 and 4T1-CXCL13 cells had no endogenous cell surface CXCR5 expression both *in vitro* and *in vivo*.

### CXCL13 expression in the 4T1 tumor microenvironment impairs the *in vivo* tumor growth and metastasis in immune-competent mice

**Figure 3.**
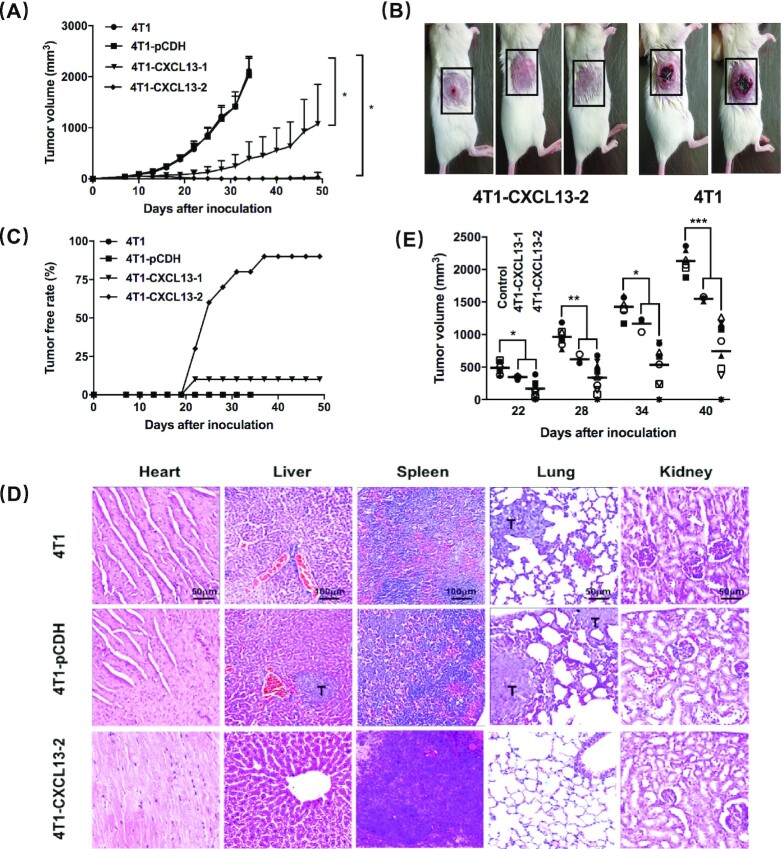
CXCL13 expression in 4T1 tumor microenvironment impaired the *in vivo* tumor growth and metastasis. (A) BALB/c mice were injected s.c. with 2 × 10^5^ 4T1, 4T1-pCDH, 4T1-CXCL13-1, or 4T1-CXCL13-2 cells, and tumors were calculated every 3 days. Bars, means ± SD (*n* = 10). This experiment was repeated 3 times (*P < 0.05). (B) Representative pictures of mice after 21 days of 4T1-CXCL13-2 (left) or parental 4T1 (right) inoculation; tumor site is shown in the black-dotted rectangle. (C) Tumor-free rate curve of each group. (D) Tumor metastases were observed in major organs by H&E pathological staining (T = tumor; lung/heart/kidney magnification, ×200; liver/spleen magnification, ×100). (E) Mice from 4T1-CXCL13-1 (*n* = 3) and 4T1-CXCL13-2 (*n* = 9) groups with >6 months complete tumor regression were rechallenged with parental 4T1 cells (2 × 10^5^), and tumor growth was significantly inhibited in both groups. The control group was naïve BALB/c mice inoculated with 2 × 10^5^ 4T1 cells (*n* = 6) (*P < 0.05, **P < 0.01, ***P < 0.001).

To evaluate the *in vivo* tumor growth, BALB/c mice were inoculated s.c. in the right dorsal flank with 4T1, 4T1-pCDH, 4T1-CXCL13-1, or 4T1-CXCL13-2 cells. The *in vivo* mouse CXCL13 expression was confirmed by immunohistochemical staining of frozen tumor tissue sections (Supplementary Fig. 1). As shown in Fig. 3A, tumor growth was significantly inhibited in 4T1-CXCL13-1 and 4T1-CXCL13-2 groups, compared with that of the parental 4T1 and 4T1-pCDH groups. No obvious difference was observed in tumor formation time and rate. Two weeks after inoculation, the tumor growth rate of 4T1-CXCL13-1 and 4T1-CXCL13-2 obviously slowed down, and on day 21, the 4T1-CXCL13-2 tumors began to shrink and were even completely eliminated (Fig. 3B). At the end of this experiment, durable tumor regression was observed in 1/10 and 9/10 of mice from the 4T1-CXCL13-1 and 4T1-CXCL13-2 groups, respectively. The remaining mouse from the 4T1-CXCL13-2 group showed stable and reduced tumor burden (Fig. 3C). Furthermore, Ki-67 staining was performed to detect the *in vivo* tumor cell proliferation. Statistical analysis of Ki-67 positive cells showed no significant difference of tumor cell proliferative between each group (Supplementary Fig. 2). Additionally, CD31 staining was performed to detect tumor angiogenesis, and significantly lower microvessel density was observed in 4T1-CXCL13-2 tumor (Supplementary Fig. 3). As for humoral immunity, tumor-specific serum antibody and antibody deposition within tumor tissue were detected. Unexpectedly, no obvious differences were observed between each group (Supplementary Fig. 4). H&E staining of mouse major organs showed no obvious pathological change or tumor metastasis in mice from the 4T1-CXCL13-2 group (Fig. 3D).

### CXCL13 elicits long-term protective antitumor immunity against parental 4T1

Furthermore, to explore whether CXCL13 can mediate long-term protection against primary tumors, 4T1-CXCL13-1 (*n* = 3) and 4T1-CXCL13-2 (*n* = 9) mice were rechallenged with parental 4T1 cells after over 6 months of complete tumor regression. Compared with the control group in which naïve BALB/c mice were inoculated with 4T1 cells (*n* = 6), significantly decreased parental 4T1 tumor growth and formation were observed in 4T1-CXCL13-1 and 4T1-CXCL13-2 tumor regression mice (Fig. 3E). Together, our data indicated that CXCL13 expression in the tumor microenvironment could significantly inhibit 4T1 tumor growth and mediate long-term protection against the parental tumor.

### The antitumor effect of CXCL13 is attenuated in immunodeficient mice

**Figure 4.**
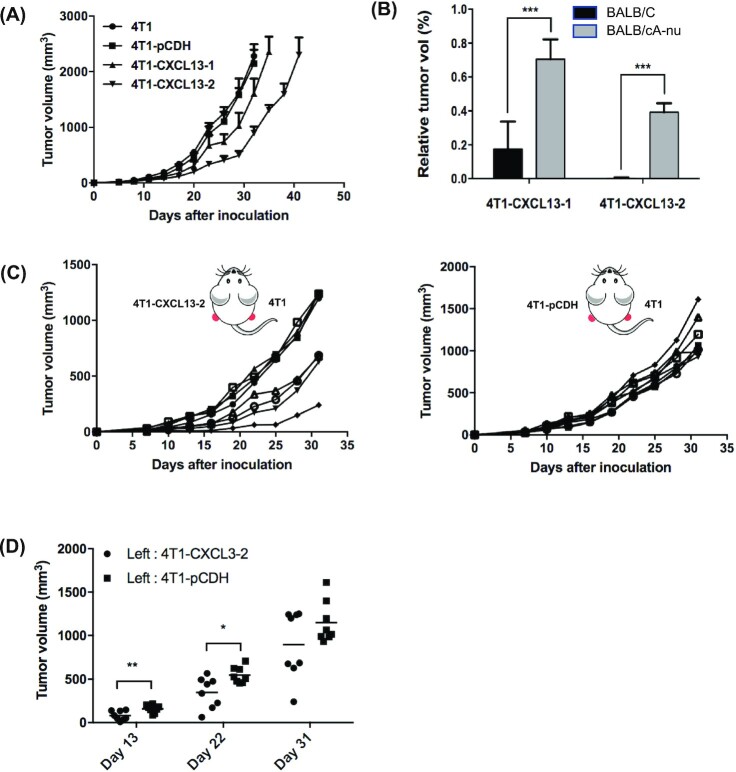
The antitumor activity of CXCL13 was mediated by both innate immunity and adaptive immunity. (A) BALB/cA-nu nude mice were injected in the right dorsal flank with 2 × 10^5^ 4T1, 4T1-pCDH, 4T1-CXCL13-1, or 4T1-CXCL13-2 cells. Bars, means ± SD (*n* = 10). (B) In BALB/c and BALB/cA-nu experiments, the ratios of average tumor volume between 4T1-CXCL13 and 4T1 groups were shown on day 32 after tumor inoculation, respectively (***P < 0.001). (C) 4T1 tumor growth in the bilateral tumor inoculation of BALB/c mice. Each curve stands for the 4T1 tumor growth of one mouse (*n* = 8). (D) Statistic results of 4T1 tumor volume on days 13, 22, and 31 after bilateral tumor inoculations (*P < 0.05, **P < 0.01).

To investigate whether CXCL13 could mediate tumor growth inhibition in immunodeficient mice, BALB/cA-nu nude mice with thymus and T-lymphocyte deficiency were inoculated s.c. with 4T1, 4T1-pCDH, 4T1-CXCL13-1, or 4T1-CXCL13-2 tumor cells. Interestingly, although compared to 4T1 and 4T1-pCDH, 4T1-CXCL13-1 or 4T1-CXCL13-2 tumors showed decreased growth rate, we did not observe complete tumor regression, which suggested attenuated antitumor activity of CXCL13 in BALB/cA-nu mice (Fig. 4A). As shown in Fig. 4B, on day 32 after inoculation, the mean relative tumor volume ratio between 4T1-CXCL13 and parental 4T1 in BALB/cA-nu model was significantly higher compared with that in BALB/c mouse model. However, the growth curve of either 4T1-CXCL13-1 or 4T1-CXCL13-2 did not coincide with that of the control groups. Considering the existence of normal NK cell effector function in BALB/cA-nu mouse, our results indicated that both innate and adaptive immunities possibly participated in CXCL13-mediated antitumor activity.

### CXCL13 expression in the 4T1 tumor microenvironment mediates rejection of distal tumor

Subsequently, to assess whether the intratumorally expressed CXCL13 elicited a systemic antitumor immune response, a bilateral tumor inoculation model was established. Two groups of BALB/c mice were simultaneously inoculated on both sides with different tumor cells: the first group was injected on the left back with 4T1-CXCL13-2 cells, and on the right with parental 4T1 cell; the second group was injected on the left back with 4T1-pCDH cells, and on the right with parental 4T1 cells. As shown in Fig. 4C, the parental 4T1 tumor growth was remarkedly decreased in 4/8 of mice from the first group, compared with that of the second group. Furthermore, on days 13 and 22 postinoculation, the average 4T1 tumor volume was significantly smaller than that of the second group (Fig. 4D). Our results indicated that the CXCL13 expressed in the 4T1 tumor microenvironment mediated a systemic antitumor immune response.

### CXCL13 expression in the 4T1 tumor microenvironment mediates enhanced immune cell infiltration

**Figure 5.**
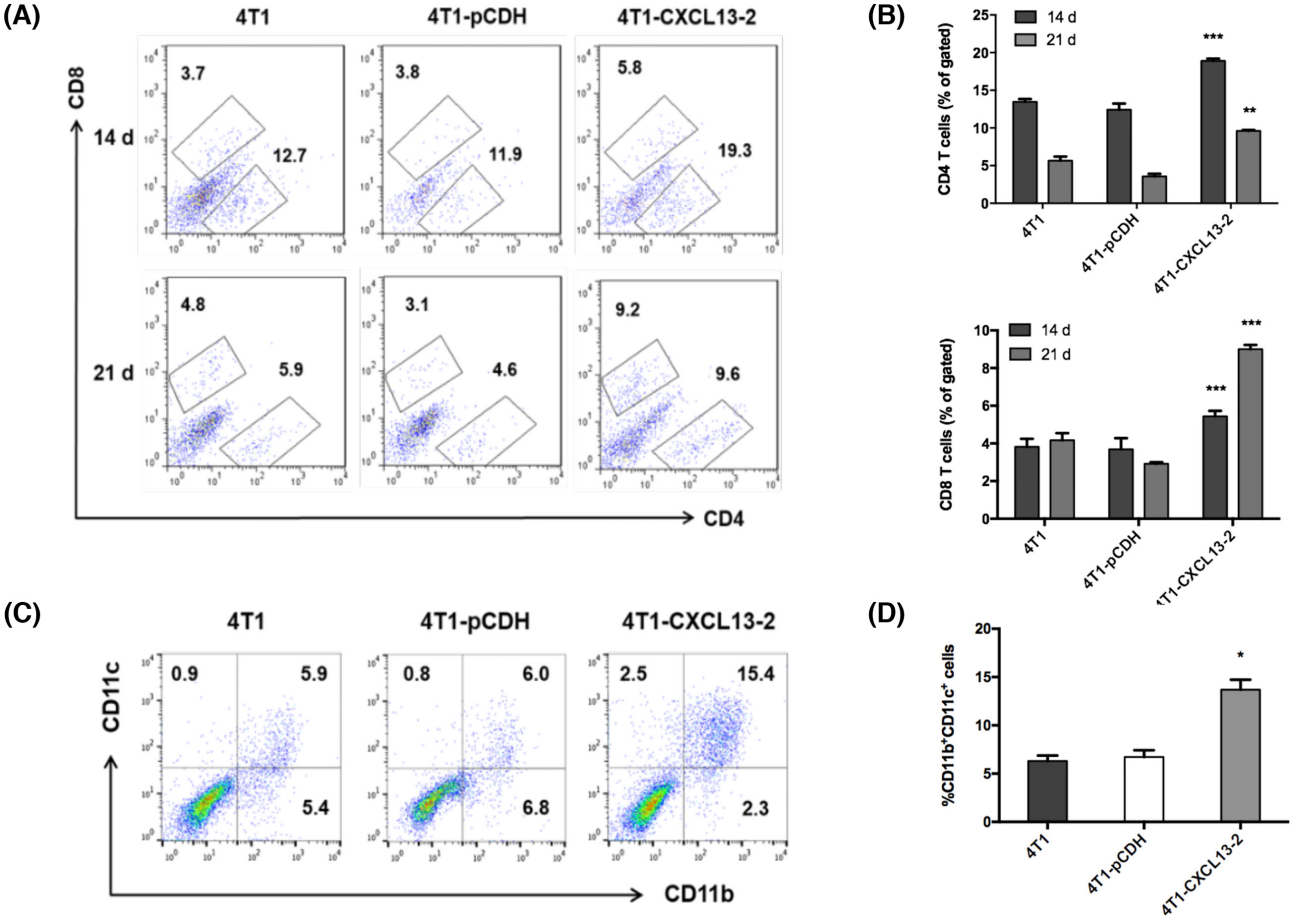
CXCL13 expression in 4T1 tumor microenvironment elicited increased infiltration of immune cells. (A) The representative flow cytometry pictures of intratumoral CD4^+^ and CD8^+^ T lymphocytes at days 14 and 21 after tumor inoculation. (B) Statistical analysis of tumor-infiltrating CD4^+^ and CD8^+^ T lymphocytes. Bars, means ± SD (**P < 0.01, ***P < 0.001, *n* = 3–4). (C) The representative flow cytometry pictures of intratumoral DC (CD11b^+^CD11c^+^) at day 21 after tumor inoculation. (D) Statistical analysis of tumor-infiltrating DC. Bars, means ± SD (*P < 0.05, *n* = 3–4).

Next, CXCL13-mediated immune cell infiltration in 4T1 tumor microenvironment was analyzed by flow cytometry. Fresh tumor tissues were harvested 14 and 21 days after inoculation in BALB/c mice, respectively, then single-cell suspension was prepared and stained for tumor-infiltrating CD4^+^, CD8^+^ T lymphocytes, and CD11b^+^CD11c^+^. Flow cytometry showed that the ratios of tumor-infiltrating CD4^+^ and CD8^+^ T lymphocytes were significantly elevated in the CXCL13-overexpressing tumor, compared with parental 4T1 and 4T1-pCDH (Fig. 5A, B). Consistently, immunofluorescence staining of tumor frozen section also validated the increased infiltration of CD4^+^ and CD8^+^ T lymphocytes (Supplementary Fig. 5). In addition, flow cytometry showed that a significantly higher proportion of CD11b^+^CD11c^+^ DC in the tumor of 4T1-CXCL13-2 group, compared to the 4T1 and 4T1-PCDH groups (Fig. 5C, D). However, unexpectedly, no obvious CD19 + B lymphocyte infiltration was observed by either immunofluorescent staining or flow cytometry (data not shown). Our results indicated that the intratumoral CXCL13 expression could induce enhanced infiltration of CD4^+^, CD8^+^ T lymphocytes, and CD11b^+^CD11c^+^ DC in the 4T1 tumor microenvironment.

### The antitumor effect of CXCL13 is impaired by immune cell depletion

**Figure 6.**
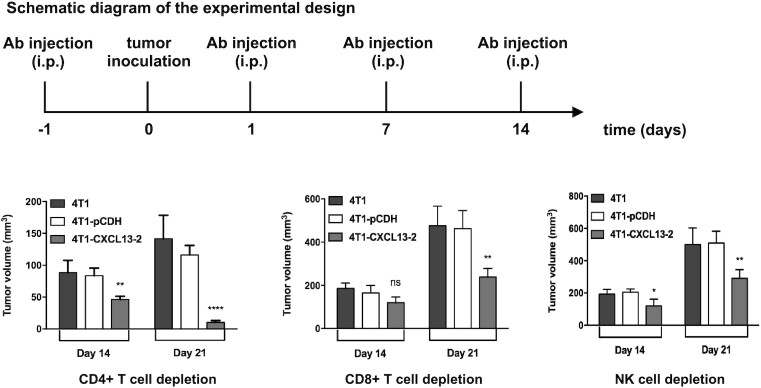
Depletion of CD4^+^ T cells, CD8^+^ T cells and NK cells impaired the antitumor effect of CXCL13. BALB/c mice were injected on the right dorsal flank with 2 × 10^5^ 4T1, 4T1-PCDH, and 4T1-CXCL13-2 cells. On days –1, 1, 7, and 14, mouse CD4 antibody, mouse CD8a antibody or mouse NK antibody was injected. Tumors were measured every 3 days starting on day 7; tumor volumes were calculated as in Fig. 6. Bars, means ± SD (*P < 0.05, **P < 0.01, ^****^P < 0.0001, *n* = 3).

To further investigate the specific immune cell type that participated in CXCL13-mediated antitumor immunity, CD4^+^, CD8^+^ T lymphocytes, and natural killer (NK) cells were depleted, respectively, in naïve BALB/c mice by consecutive i.p. injections of antibodies. Consequently, on day 21 after tumor cell inoculation, the tumor volume of 4T1-CXCL13-2 in CD4^+^ T lymphocyte depletion mice showed similar shrinkage and regression as in previous BALB/c mice experiment, whereas CXCL13-mediated tumor inhibition was almost completely abolished after CD8^+^ T lymphocyte depletion. In mice depleted with CD8^+^ T cells, the tumor volume of 4T1-CXCL13-2 remarkedly increased from day 14 to day 21. Similarly, this was also observed in NK depletion mice, although to a lesser degree (Fig. 6). Together, our data indicated that CXCL13-mediated antitumor immunity could be primarily mediated by CD8^+^ T lymphocyte and NK cells.

## Discussion

To date, CXCL13 has been widely accepted as an important mediator in the initiation, progression, and metastasis of solid tumors, and identified as one of the most strongly overexpressed chemokines in breast cancer. However, its exact role in breast cancer remains controversial. While some studies provided evidence that high intratumoral CXCL13 expression was associated with a favorable prognosis and had an independent influence on patient survival,^11,19^ others showed opposite results.^18,26^ In the current study, we found that mouse TNBC 4T1 transfected to overexpress CXCL13 showed significant *in vivo* tumor growth inhibition in immune-competent mice, and at the same time, mediated long-term protective antitumor immunity and rejection of distal parental tumor. Of note, no obvious *in vitro* or *in vivo* cell surface CXCR5 expressions were detected in parental 4T1 or 4T1-CXCL13 strains. To our knowledge, this study is the first to investigate the immune regulation effect of intratumorally expressed CXCL13 in breast cancer.

Human cancers take advantages of a complex chemokine network to directly and indirectly affect tumor growth, survival, migration, and angiogenesis.^27^ Therefore, understanding the intricate roles of chemokine systems in cancer is important for better clinical decision making. In fact, several CXC chemokines have been reported to play positive roles in antitumor immunity. For instance, CXCL9 and CXCL10 secretions by Ewing sarcoma chemoattracted a large number of CD8^+^ cytotoxic T lymphocytes and were negatively correlated with tumor progression.^28^ In the current study, we found that the intratumoral CXCL13 expression could mediate significant *in vivo* growth inhibition in mouse 4T1 breast cancer, and 4T1-CXCL13 tumors started to shrink and were even completely eliminated at ∼3 weeks after inoculation. This result was consistent with one previous study in which tumor rejection in an orthotopic colon cancer model was observed in 80% (4/5) of mice at weeks 2.5 and 3 after recombinant CXCL13 injection.^29^

Immune cell infiltration in TNBC has been considered to be correlated with a favorable clinical outcome.^30^ Meanwhile, high-expressing CXCL13 tumors have also been identified with a higher TIL density.^11,31^ Furthermore, after transfection with the transcription factor of CXCL13, human breast cancer MDA-MB-231 showed special recruitment to healthy donor-derived CXCR5 + T and B cells.^23^ Therefore, CXCR5 + immune cells can probably respond to the chemotaxis signal of CXCL13, migrate to tumor microenvironment, and elicit antitumor immune response. In this study, we observed increased infiltrations of CD4^+^, CD8^+^ T lymphocytes, and CD11b^+^CD11c^+^ DC in a CXCL13-expressing 4T1 tumor microenvironment. Unexpectedly, increased B lymphocyte infiltration was not observed, which was most likely due to the sparse B cell infiltration in the s.c. model of 4T1 cells. In fact, CXCR5 has been reported to express on multiple immune cells, including B lymphocytes, ^23^ a subset of CD4^+^,^32^ CD8^+^ T lymphocytes,^33^ dermal-type DCs,^34,35^ as well as NK cells.^36^ We also found in BALB/cA-nu mice that lack of mature T cells, CXCL13-mediated tumor growth inhibition was significantly attenuated but not completely abrogated, indicating potential participation of innate immune cells. Long-term protective immunity against parental 4T1 tumor was observed in 4T1-CXCL13 tumor regression mice. Our further investigation revealed that the antitumor activity of CXCL13 was markedly diminished after CD8^+^ T lymphocyte or NK cell depletion, which was consistent with the previous finding that activation of cytotoxic T lymphocytes and NK cells were both important for protective tumor immune surveillance. ^37^ CD8^+^ T lymphocyte infiltration in breast cancer was correlated with good clinical prognosis,^38,39^ especially in TNBC.^40^ Together, our results indicated that CXCL13 expression in the 4T1 tumor microenvironment triggered both innate and adaptive antitumor immune responses.

Despite this, previous studies reported that CXCL13 expression in human breast cancer promoted tumor cell growth, migration and LNM.^22,41^ It has also been shown that after CXCR5 transfection, human breast cancer cells had significantly upregulated mesenchymal markers and EMT regulators when stimulated with recombinant CXCL13.^22^ However, in this study, CXCL13-overexpressing 4T1 cells exhibited unaltered proliferation activity. Therefore, tumor cell surface CXCR5 was evaluated by flow cytometry in this study. As expected, parental 4T1 and 4T1-CXCL13 cells showed no obvious CXCR5 expression both *in vitro* and *in vivo*, which can probably explain the previously contradictory findings of CXCL13 in breast cancer. In fact, there was accumulating evidence that high CXCL13 expression in breast cancer tissue was related with good clinical prognosis, and tumor-expressing CXCL13 was considered an independent influence factor for breast cancer patient survival, especially in TNBC.^19^ In addition to cancer cell-secreting CXCL13, tumor-infiltrating T lymphocytes and DCs have also been considered as the main source of CXCL13, typified by CXCL13^+^/CD4^+^ follicular helper T cells^32,42^ and follicular dendritic cells.^43^ The infiltration of CXCL13^+^ immune cells in the breast cancer microenvironment has also been reported to predict good clinical good prognosis.

The complex CXCL13–CXCR5 intratumoral interaction network contains both autonomous and nonautonomous responses to cancer cells, which may further vary depending on the specific cancer types, especially CXCR5 expression status on the tumor cell surface.^44^ When the tumor cell expresses CXCR5 itself, CXCL13 can directly act on cancer cells to promote proliferation, migration, and invasion. When the tumor cell has no endogenous CXCR5 expression, CXCL13 secretion in the tumor microenvironment can chemoattract CXCR5^+^ immune cells and trigger antigen-specific antitumor immunity. Therefore, when considering the specific effect of CXCL13 in the tumor microenvironment, attention needs to be paid to the expression status of CXCR5 on cancer cells. A potential limitation of this study is that the phenotype analysis of tumor-infiltrating lymphocytes was hindered by the sparse immune cell infiltration in the 4T1 s.c. tumor model, and further investigation is required in the future. Moreover, we observed significantly lower microvessel density in CXCL13-overexpressing 4T1 tumor, and this was supported by one previous study that CXCL13 modulated angiogenesis by inhibiting fibroblast growth factor 2-induced chemotaxis, proliferation, and survival of endothelial cells.^45^

In conclusion, our study provided evidence that CXCL13 expression in a mouse 4T1 tumor microenvironment triggered effective antitumor immunity by chemoattracting immune cell infiltrations, and CXCL13 could been considered as a novel prognostic marker for TNBC. Due to the complexities of the CXCL13/CXCR5 axis in tumor milieu, CXCR5 expression status on tumor cells should be carefully assessed in clinical practice.

## Supplementary Material

pbab020_Supplemental_File
