# Supplementary material for: CXCL13 expression in mouse 4T1 breast cancer microenvironment elicits antitumor immune response by regulating immune cell infiltration
Source: Precis Clin Med. 2021 Aug 4;4(3):155–67. doi: 10.1093/pcmedi/pbab020 (PMC8982548; doi:10.1093/pcmedi/pbab020)
Supplement: pbab020_Supplemental_File [file pbab020_supplemental_file.doc]

**Supplementary Figure 1. Immunohistochemistry (IHC) detection of CXCL13 expression.**

Tumor tissues were obtained at necropsy in BALB/c mice, and frozen sections were stained to detect CXCL13 expression. Magnification, ×200.

**Supplementary Figure 2.** **Immunohistochemistry (IHC) detection of Ki-67 expression in tumor tissues.** Tumor tissues were obtained at necropsy in BALB/c mice, and formalin-fixed paraffin-embedded sections were stained for Ki-67 expression. Magnification, ×200.

**Supplementary Figure 3. Immunohistochemistry (IHC) detection of CD31 expression.** Tumor tissues were obtained at necropsy in BALB/c mice, and frozen sections were stained for CD31 expression. Magnification, ×200.

**Supplementary Figure 4.** **Tumor-specific antibody detections.** The upper figures show the immunohistochemistry (IHC) staining of IgG deposition in tumor tissues. The lower figures show the flow cytometry detection of tumor-specific IgG in serum. Parental 4T1 cells were incubated with serum (1:100 dilution) of different groups of mice, and then stained with FITC-labeled goat anti-mouse IgG antibody. Magnification, ×200.

**Supplementary Figure 5. Immunofluorescent staining of tumor-infiltrating T lymphocytes.** Tumor tissues were obtained at necropsy in BALB/c mice, and frozen sections were stained for CD4+ and CD8+ T cells. Magnification, ×500.

**Supplementary Figure 6. Ancestry gating principles of flow analysis.** The percentages of the different immune cell subpopulations were gated and defined based on forward and sider scatter (FSC/SSC) plots. (A) The representative flow cytometry gating pictures of intratumoral CD4+ and CD8+ T lymphocytes. (B) The representative flow cytometry gating pictures of intratumoral DC (CD11b+CD11c+).
